# A perspective on physiological studies supporting the provision of scientific advice for the management of Fraser River sockeye salmon (*Oncorhynchus nerka*)

**DOI:** 10.1093/conphys/cow026

**Published:** 2016-08-26

**Authors:** David A. Patterson, Steven J. Cooke, Scott G. Hinch, Kendra A. Robinson, Nathan Young, Anthony P. Farrell, Kristina M. Miller

**Affiliations:** 1 Fisheries and Oceans Canada, Science Branch, Cooperative Resource Management Institute, School of Resource and Environmental Management, Simon Fraser University, Burnaby, BC, Canada V5A 1S6; 2 Fish Ecology and Conservation Physiology Laboratory, Department of Biology and Institute of Environmental Science, Carleton University, Ottawa, ON, Canada K1S 5B6; 3 Pacific Salmon Ecology and Conservation Laboratory, Department of Forest and Conservation Sciences, University of British Columbia, Vancouver, BC, Canada V6T 1Z4; 4 Department of Sociology and Anthropology, University of Ottawa, Ottawa, ON, Canada K1N 6N5; 5 Department of Zoology and Faculty of Land and Food Systems, University of British Columbia, Vancouver, BC, Canada V6T 1Z4; 6 Fisheries and Oceans Canada, Science Branch, Pacific Biological Station, 3190 Hammond Bay Road, Nanaimo, BC, Canada V9T 6N7

**Keywords:** Migration mortality, scientific advice, sockeye salmon, thermal physiology

## Abstract

The inability of physiologists to effect change in fisheries management has been the source of frustration for many decades. Close collaboration between fisheries managers and researchers has afforded our interdisciplinary team an unusual opportunity to evaluate the emerging impact that physiology can have in providing relevant and credible scientific advice to assist in management decisions. We categorize the quality of scientific advice given to management into five levels based on the type of scientific activity and resulting advice (notions, observations, descriptions, predictions and prescriptions). We argue that, ideally, both managers and researchers have concomitant but separate responsibilities for increasing the level of scientific advice provided. The responsibility of managers involves clear communication of management objectives to researchers, including exact descriptions of knowledge needs and researchable problems. The role of the researcher is to provide scientific advice based on the current state of scientific information and the level of integration with management. The examples of scientific advice discussed herein relate to physiological research on the impact of high discharge and water temperature, pathogens, sex and fisheries interactions on in-river migration success of adult Fraser River sockeye salmon (*Oncorhynchus nerka*) and the increased understanding and quality of scientific advice that emerges. We submit that success in increasing the quality of scientific advice is a function of political motivation linked to funding, legal clarity in management objectives, collaborative structures in government and academia, personal relationships, access to interdisciplinary experts and scientific peer acceptance. The major challenges with advancing scientific advice include uncertainty in results, lack of integration with management needs and institutional caution in adopting new research. We hope that conservation physiologists can learn from our experiences of providing scientific advice to management to increase the potential for this growing field of research to have a positive influence on resource management.

## Abstract (precis)

Science advice based on physiology is supporting harvest decisions for sockeye salmon by providing a mechanistic understanding for in river mortality. This success is a function of political will, clarity in management objectives, and science management integration. Uncertainty in results and institutional caution are major challenges for using science advice.

## Introduction

The recent expansion of knowledge in the field of conservation physiology (see Wikelski and Cooke, 2006; Cooke *et al*., 2013; Lennox and Cooke, 2014) has led to a growing recognition by researchers and managers alike of the potential benefit that physiological studies can have in addressing some of the key challenges in aquatic conservation (Young *et al*., 2006; Horodysky *et al*., 2015). The major advantage of applying physiological principles to management problems is the more complete mechanistic understanding (i.e. cause and effect) that traditionally comes from physiology (Cooke and O'Connor, 2010; Horodysky *et al*., 2015). Therefore, a defensible physiological explanation can be desired by managers to help inform decisions that have previously been based on simple correlative associations of different stressors and fish survival derived mainly from ecological studies (e.g. Macdonald *et al*., 2010). The challenge for researchers is the paucity of information on how best to transfer physiological research at the individual level to scientifically defensible predictions of population-level consequences for aquatic organisms that are desired by managers (Coristine *et al*., 2014).

Much has been written about how different scientific disciplines create knowledge (Knorr Cetina, 1999; Becher and Trowler, 2001). In the life sciences, this process usually begins with a simple idea that, if deemed sufficiently interesting and promising, is refined using basic observations and preliminary research, further developed by testable hypotheses and in-depth descriptions through to quantifiable predictions, and finally, worked into general principles that allow us to make confident assertions about nature. At each step, the idea may be abandoned or changed, and very few scientific ideas evolve into general principles widely accepted by peers (Latour, 1987).

**Figure 1:**
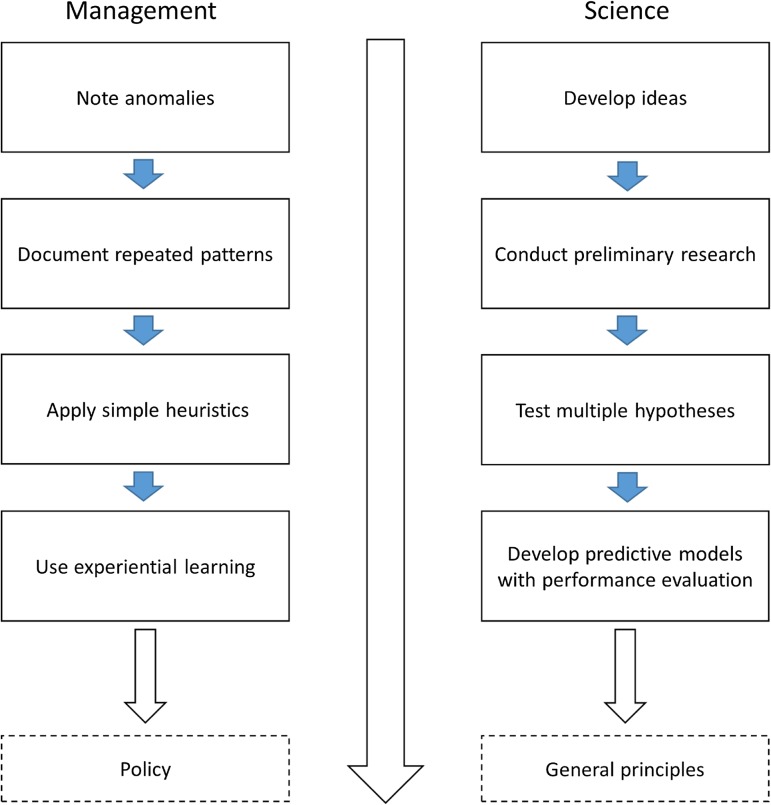
The learning methods used by managers and researchers that demonstrate the different and independent parallel activities designed to serve the information needs of their respective vocations. For management, the information is used for decision-making purposes and can lead to broad policy statements. For scientists, the information is used to increase scientific understanding that can potentially lead to general principles about the natural world. This is not meant to imply that managers do not use scientific learning methods as well.

Less familiar to researchers is the parallel, stepwise progression that fisheries managers commonly use ‘to learn’ about their environment in order to make informed decisions and develop policies (Fig. 1). For managers, the corollary process involves noting anomalies, documenting repeated patterns, articulating the management problem and surveying for solutions, defining the management objectives and constraints in relationship to the problem, communicating the potential mitigation measures with interested parties and developing general policy statements to cover future related issues. Managers often use different heuristics to find solutions, such as experiential learning and, as such, they do not explicitly need to engage in the scientific process to assist them (Fig. 1; Pullin *et al*., 2004). In order to begin to understand the knowledge-transfer challenges faced by researchers, it is important to note that these different learning methods used by managers and researchers can be completely independent, parallel processes that are designed to serve the needs of their respective vocations (Young *et al*., 2013).

Researchers and managers are embedded in very different institutional environments, with different mandates, pressures and reward systems. Social science research has shown that scientists are most influenced by their community of peers. Career rewards in the form of research funding, promotions, reputation and influence are based on the system of peer review; a system that encourages scientists to speak predominantly amongst themselves and to communicate in ways that are most useful to peers rather than potential outside users of their knowledge and findings. Managers, on the contrary, are judged on their successful handling of real-world problems that involve a wide range of other actors, including politicians, bureaucrats, conservation groups, indigenous groups holding special rights and other users of the resources. In this complex institutional environment, managers draw on multiple forms of social and ecological knowledge, including personal and collective experience, professional judgement, intuition and direct observation, as well as scientific models and data (Pullin *et al*., 2004; Fazey *et al*., 2006). We therefore cannot assume that new science will ‘trickle down’ to management decision-making simply because of its quality or (presumed) relevance. Instead, we argue that better communication and coordination between researchers and managers can be achieved by integrating the stepwise pathways for increased understanding that we illustrate in Fig. 1.

**Figure 2:**
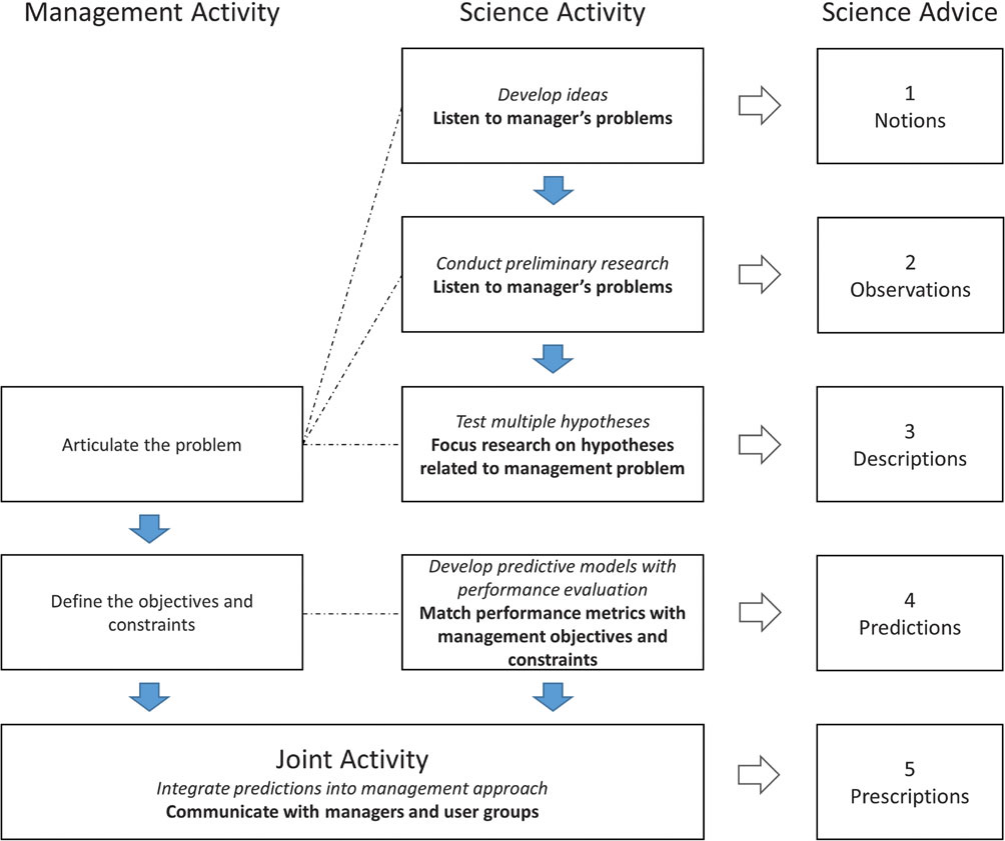
The idealized integration of management and scientific activities for the provision of different levels of scientific advice to aid decision-making. The dashed lines represent the communication between managers and researchers that is necessary to promote the advancement in the level of scientific advice provided (see ‘*Communication*’ section). The diagram is presented from the researcher's perspective of trying to advise managers. The level of scientific advice is a function of both the state of scientific knowledge determined by the scientific learning activity (denoted in italics) and the specific integration activity with management (denoted in bold). For example, prescriptions (i.e. Level 5 scientific advice) result from a combination of high-quality science (i.e. predictions) and joint communication activities with managers and affected user groups.

Scientific advice can be given to aid in the development of management policy or, more commonly, it can be given to aid in specific management decisions. The latter is the focus of this review. We have limited our discussion of scientific advice to the provision of scientifically defensible, transparent and reproducible information that can help to inform specific management decisions. Based on our experiences of providing scientific advice to aid in decision-making for management, we propose an idealized model of generating scientific advice using five levels that parallel the stages of learning activities and knowledge acquisition for management and science illustrated in Fig. 1. Each scientific advice level represents an increase in the veracity of the scientific advice. This continuum starts with Level 1 advice that provides simple educated thoughts about how the world might work and proceeds through to Level 5 advice that generates prescriptions using quantifiable predictions of outcomes that can be directly used in management decisions. We have modified Fig. 1, which illustrates how both groups seek to acquire information, reducing uncertainty and gaining insight, to reflect a more co-dependent activity relationship that can generate quality scientific advice (Fig. 2). Figure 2 presents the different levels of scientific advice that can result once a management problem can be posed accurately as a scientific question. The ability of science to respond at a given scientific advice level will depend on the state of knowledge and connectivity to management. The level of scientific advice is defined from the scientific perspective of increasing scientific defensibility (transparency, repeatability, strength of evidence in quantity and consistency) and reducing uncertainty (predictive power), as well as increasing the potential utility within management (level of integration with management problems, objectives and constraints). The communication pathways and management activities that facilitate connectivity are illustrated to emphasize that advancement beyond Level 3 scientific advice requires a more co-dependent relationship (Fig. 2).

There are an increasing number of examples of conservation physiology being used in the development of scientific advice for resource management (e.g. Cooke *et al*., 2012; Madliger *et al*., 2015), but there are very few details on the process and difficulties associated with integrating physiological results with scientific advice and management needs. In response, we provide our perspective of the overall integration process by providing detailed examples of successful and unsuccessful attempts at applying conservation physiology to fisheries management problems from our work. More specifically, we have drawn examples from our biological research to communicate the emerging role that physiology is having in raising the level of scientific advice being given to aid the management of Fraser River sockeye salmon (*Oncorhynchus nerka*) fisheries. This iconic salmon species is arguably the most culturally important and well-studied Pacific salmon in Canada (reviewed by Hinch *et al*., 2012; Johnson *et al*., 2012). The remarkable anadromous migrations of semelparous adult sockeye salmon have long fascinated physiologists independent of an explicit management problem, with early research being focused on energetics (Idler and Clemens, 1959; Brett, 1965), homing ability (Fagerlund *et al*., 1963) and senescence (McBride *et al*., 1963; Fagerlund, 1967; Donaldson and Fagerlund, 1970). More recently, the uncertainty surrounding the causes of declines in Fraser River sockeye salmon abundance caught national political attention, resulting in a major $30+ million federal judicial review called by the Prime Minister of Canada (Cohen, 2012). This latest judicial review is part of the long history of political interest in the migration problems of Fraser River sockeye salmon, starting with the Hells Gate slide (e.g. Ricker, 1947; Larkin, 1992; Fraser, 1995; Williams, 2005). It is against this interesting scientific and long-standing political background that our group focused our physiological research on major factors that can impact the upstream migration success of adult sockeye salmon returning to spawn in the Fraser River, with an aim to provide scientific advice relevant to the conservation of this important population.

In the first part of this paper, we conduct an historic review of the biological research on the impacts of adverse environmental conditions (high discharge and high water temperature) on in-river mortality of Fraser River sockeye salmon. The aim is to characterize the five levels of scientific advice and to showcase the recent emergence of physiology in connection with the quality of scientific advice. This recap identifies the conditions in the management and science realms that we associate with success in providing scientific advice at higher levels.

In the second part of this paper, we discuss the current scientific advice given to management based on the physiological research into the role of pathogens, sex and fisheries interactions in understanding in-river mortality. The purpose of the second section is to showcase both the breadth of success in using conservation physiology and the current challenges for advancing the utility of this information for management. Our goal is to provide other conservation physiologists with an understanding of the steps that are likely to be involved in gaining management uptake of their research, based on our successes and challenges using the rich history of biological and physiological research conducted on Fraser River sockeye salmon. We recognize that local geopolitical, sociocultural and institutional norms will influence the extent to which the specifics described here apply, but we submit that the lessons learned and general approach described herein should be of broad relevance to those working in different jurisdictions on different species and issues.

## Part I: levels of scientific advice for in-river mortality

This section focuses on the historical progression of scientific advice related to water temperature and discharge that has been provided to management in response to high in-river mortalities of Fraser River sockeye salmon. For each level of scientific advice, we describe the type of scientific activity that has occurred and the resulting scientific advice that has been provided to managers through time. We also discuss the scientific and management activities that facilitate a higher level of scientific advice. These examples help to characterize our idealized model of scientific advice (Fig. 2).

The earliest written records of run failure associated with Fraser River sockeye salmon date back to catch records from the Hudson Bay Company in the 1800s (Cooper and Henry, 1962). Although managers took note of such anomalies, they did not make any connection to specific environmental conditions, such as high discharge or water temperatures. The first series of notable run failures associated with environmental conditions for Fraser River sockeye salmon occurred in the early 1900s, with a series of major rockslides resulting from railroad construction in the Fraser Canyon at Hells Gate. This culminated in effectively delaying and, in some cases, completely blocking upstream migration for large numbers of migrating sockeye salmon (Thompson, 1945). The collapse of the record 1913 cohort of sockeye salmon 4 years later (catch declined from 32 million in 1913 to 7 million in 1917) was further confirmation of a major problem for management to consider (Roos, 1991). Since then, it has been recognized that a variable portion of Fraser River sockeye salmon that are estimated to enter the lower Fraser River are not accounted for at the spawning grounds after adjusting for estimates of in-river harvest. The source of this discrepancy is a combination of assessment errors in estimates of catch, and lower and upper-river estimates of abundance, as well as natural in-river mortality (Patterson *et al*., 2007b).

Estimates of these discrepancies over the past 20 years have a cumulative total net loss of 18 million fish. In comparison, 80 million fish were harvested and 70 million escaped to spawn during the same time period (Pacific Salmon Commission, unpublished data). These in-river losses represent substantial foregone opportunities for First Nation communities to access salmon for food, social and ceremonial purposes, limitations for angler participation, millions of dollars in lost revenue to the salmon fishing industry, reductions in spawners for other ecosystem values, and loss of future recruitment. Therefore, in-river mortality associated with adverse migration conditions provides a clear problem, for which researchers can provide scientific advice on likely factors and mechanisms contributing to mortality and, thus, speak to the efficacy of potential mitigation measures. We will describe some of the research and information given at different levels of scientific advice over the years in relationship to in-river mortality.

### Level 1: notions

The first level of scientific advice, and lowest level of veracity, in response to a management problem involves the simple generation of ideas based on the researcher's current knowledge base. This brainstorming is done without any direct empirical testing, before providing their best ‘notions’ to management, and is therefore subject to the potential cognitive frailties associated with any expert opinions (Sutherland and Burgman, 2015). We are not privy to the preliminary ideas that researchers shared (if any) with managers regarding the impact of environmental conditions on in-river mortality of Fraser River sockeye salmon in response to the first records of run failures in the 1800s. We speculate that any notions, if shared with management, had minimal impact on decisions, given that they are not recorded in any historic management documents.

### Level 2: observations

The next level of scientific advice requires researchers to formulate a testable hypothesis regarding their ideas and start fundamental (also known as basic or discovery) research. The scientific advice at Level 2 is based on the scientific ‘observations’ derived from this basic research. In our example, observations of delays and downstream mortalities in the Fraser Canyon were meticulously recorded by field experts during the major rock slides from 1911 to 1914 (Thompson, 1945). It was theorized that the extreme hydraulic challenges created by the rock slides exceeded the swimming ability of most of the upstream-migrating sockeye salmon (Talbot and Jackson, 1950). This led fisheries biologists who worked for the management agencies responsible for the fisheries to test ideas regarding swimming performance and to assess passage ability directly using large-scale tagging projects (Thompson, 1945; Talbot and Jackson, 1950). Interestingly, not all fisheries scientists were in agreement with respect to the central role that migration barriers at Hells Gate had in limiting the productivity of stocks upstream of the barrier. Ricker (1947) had serious reservations about the quality of the tagging studies to the extent that he questioned the primacy of hydraulic barriers over fishery exploitation rates as being the primary cause for depressed sockeye salmon stocks. However, based on Thompson's (1945) interpretation of this preliminary research, management expeditiously responded by constructing fish passage facilities at Hells Gate in the late 1940s. The magnitude of the response by management to Level 2 scientific advice shows that even preliminary or contested research can have a high degree of impact on management decisions, but instances such as this are rare and usually applied at a small scale or a single site so that management can assess its effectiveness before widespread application (Gross, 2010).

Prior to the 1960s, there is almost no explicit mention of thermal impacts in relationship to adverse migration conditions for sockeye salmon (Foerster, 1968) even though physiologists had long since recognized temperature as a principal factor in controlling biological processes and ultimate survival in fish (Fry, 1947). In Foerster's (1968) comprehensive review of sockeye salmon research and management, he mentions only 2 years with reports of in-river mortality, 1942 and 1958. Only the former event was linked explicitly to high temperatures, despite the fact that the latter was one of the warmest years on record for the Fraser River (Patterson *et al*., 2007a). The 1958 in-river loss estimate was 7.9% of the total run, considered above average at the time and enough for management to report in the annual summaries (IPFSC, 1959), but insufficient to warrant a management response. At this point, any scientific advice associated with thermal physiology would probably have triggered the curiosity of management. However, without an obvious link to a specific management problem (see proposed connectivity in Fig. 2), such as repeated observations of high mortality events with high water temperature, and with an absence of direct physiological research on adult sockeye salmon thermal tolerance, it probably would have had a minimal impact.

### Level 3: descriptions

The third level of scientific advice is founded on the research results from more scientifically rigorous studies. This requires the researcher to work on generating biologically based descriptive models related to understanding the problem as identified by management and to present these model outputs (i.e. ‘descriptions’) to managers as scientific advice. In the case of the impacts of environmental conditions on in-river migration mortality, the continued challenge for researchers was to seek robust, scientific explanations for these in-river losses. In the 1960s, it became clear that high water temperatures, in addition to high discharge, during in-river migration were linked to natural mortality. It was the researchers within the agencies responsible for managing the fisheries who started a lot of the work on water temperature impacts on sockeye salmon physiology, examining the connections to disease (Colgrove and Wood, 1966; Williams, 1973), swimming ability (Brett, 1965) and upper thermal limits (Servizi and Jensen, 1977). This latter and oft-cited work on thermal tolerance was in direct response to a management request to determine the thermal impact of a major water diversion project in the Fraser River watershed. Unfortunately, the findings from this work probably set back further research on thermal physiology for several decades because of misinterpretation of the scientific results on the ecological relevance of upper lethal thermal limits. Further work on the impacts of high discharge conditions continued to stay ahead of temperature impacts.

The involvement of our group with the research on Fraser River sockeye salmon migration problems in the 1990s was initially focused on high discharge and not thermal physiology. The aim was to describe swimming performance (Hinch *et al*., 1996), variation in swimming behaviour (Hinch and Rand, 1998) and, ultimately, migration survival (Rand and Hinch, 1998; Hinch and Bratty, 2000) associated with the hydrological challenges of the Fraser River. This work provided physiological evidence to explain why certain sections of the Fraser River were more challenging for fish to ascend than others, moving beyond unreliable observations of carcasses (Patterson *et al*., 2007b) and weak correlations of discharge with in-river loss estimates (Macdonald, 2000) to gain a better mechanistic understanding of in-river mortality. These physiological descriptions of the impact of high discharge on sockeye salmon were provided to management and influenced some of the early harvest decisions regarding adverse migration conditions (Macdonald, 2000; Macdonald *et al*., 2000). Moreover, the modelling of energy expenditures in relationship to encounter velocities led to more physiological research on the role of water temperature in salmon migration metabolism, an essential component of bioenergetics modelling for poikilotherms (Rand *et al*., 2006).

Today, the direct and indirect physiological impacts of high water temperature comprise a large portion of the Level 3 scientific advice given to managers regarding the factors that impact in-river survival of Fraser River sockeye salmon. This research on thermal physiology has developed from historic pattern recognition of increasing high mortality events associated with high migration temperatures (Gilhousen, 1990) to a focus on elucidating physiological mechanisms that will reduce the uncertainty associated with understanding thermal-based mortality in wild salmon. Our group has been studying the myriad of ways that water temperature is impacting survival by measuring a variety of physiological responses, including swimming ability and behaviour (MacNutt *et al*., 2006), cardiorespiratory performance (Farrell *et al*., 2008; Eliason *et al*., 2011, 2013), disease progression (Wagner *et al*., 2005; Crossin *et al*., 2008; Mathes *et al*., 2010), genomic and cellular responses (Jeffries *et al*., 2012a, b) and metabolism (Clark *et al*., 2010). The overall scientific advice from this descriptive work is consistent; high water temperature and high discharge have a negative impact on many aspects of salmon physiology and, ultimately, the survival of sockeye salmon (reviewed by Cooke *et al*., 2012; Hinch *et al*., 2012). Managers are now well informed regarding the physiological impacts of high temperature and high discharge and can consider this information to account for in-river losses. In addition, the stage is set to move these descriptive research results to Level 4 scientific advice by aligning management objectives with predictive models that relate environmental conditions to in-river mortality.

### Level 4: predictions

The fourth level of scientific advice presents predictions of different outcomes to aid management in decision-making. This means moving from descriptive models focused on an improved biological understanding to predictive models, complete with an estimate of uncertainty (Harwood and Stokes, 2003; Ascough *et al*., 2008). Ideally, this is a combination of more strategic research and different analytical approaches on the part of the researcher, and more precise feedback from managers regarding their objectives and constraints. Management feedback is required to provide a more thorough description of the fishery, including the following: the legal framework, i.e. defining the management actions the agency have regulatory control over (e.g. the spatial location of fisheries); the goals, i.e. articulating the objectives of the fishery (e.g. the alternative goals of maximizing total harvest vs. fishing opportunity); and the operational constraints, i.e. communicating the practical limitations of executing a fishery (e.g. the lead time required to open or close a fishery). Management needs to determine how the results generated from a quantitative model that can predict particular outcomes could be used under the existing operational and regulatory constraints.

For Fraser River sockeye salmon, a major reason why the physiological research on the impacts of adverse migration conditions is currently used to support harvest management decision lies in the clarity of how the information fits into the overall management process. There is a clear legal mandate laid out in the 1999 Pacific Salmon Treaty (bilateral agreement between Canada and USA), in Article VI, Annex IV, Chapter 4 (10), to prevent overfishing by both countries. The treaty states that spawning escapement goals are a clear management objective of the agreement, and there is a clear mechanism to incorporate scientific advice into the process, as stated in Article VI, Annex IV, Chapter 4 (13b): ‘incorporate … management adjustments [harvest changes] that deal with environmental conditions [discharge, temperature] during in-river migration that could significantly impact the Fraser River Panel's ability to achieve spawning escapement objectives’ (Pacific Salmon Treaty, 1999). This legal background has helped to inform how the research is conducted and the types of questions asked in the development of both descriptive and predictive models.

The descriptive models used in generating Level 3 scientific advice are about understanding biological relationships between adverse migration conditions and in-river mortality. The model selection criteria typically rely on biological realism (i.e. physiological support), model fit and model sensitivity. The main target audience for descriptive models is other researchers (i.e. for primary publication), whereas the predictive models, such as those described herein, are built for applied management purposes. As such, the developers of predictive models that forecast events for managers have to consider some key additional features in model selection, including model predictive power (e.g. bias and precision), forecasting constraints for predictor variables (e.g. water temperature and discharge forecasting) and management constraints (e.g. lead time to adjust fish harvest). The last of these features reflects the fact that predictive models are built to predict the outcome of different management responses within a realistic set of conditions. In the case of Fraser River sockeye salmon, in-season harvest adjustment models that use water temperature as a predictor variable must rely on forecasted temperatures (Hague and Patterson, 2014). This is to allow time to adjust harvest that normally occurs seaward (i.e. downstream and earlier) of the potentially damaging high water temperatures that the fish would subsequently experience in the river. In simple terms, throughout the fishing season the water temperature and discharge are forecasted, an estimate of loss is predicted by the models that use the forecasted environmental conditions, and harvest can be adjusted according to the expected losses (Hague and Patterson, 2007). These quantitative models that use water temperature to forecast in-river mortality rely on physiology as the primary rationale (Macdonald *et al*., 2010). The models work by quantifying the historic relationship between in-river loss and different metrics of water temperature and discharge. The water temperature metrics that are used reflect both mean temperature exposure and the threshold responses to high temperatures. The former is justified by physiological research on energy expenditures and disease progression (e.g. Wagner *et al*., 2005; Rand *et al*., 2006; Crossin *et al*., 2008), and the latter is supported by research on aerobic scope and cardiac failure (e.g. Farrell *et al*., 2008; Mathes *et al*., 2010; Eliason *et al*., 2011). Today, fisheries management is presented with an estimate of loss, with sufficient lead time to adjust harvest and proactively change the probability associated with achieving escapement goals (Macdonald *et al*., 2010), with the knowledge that the scientific advice is supported by internationally recognized physiological research.

### Level 5: prescriptions

This fifth level of scientific advice requires the development of scientific prescriptions as part of an integrated management approach. At Level 4, researchers have collated and provided a synthesis of their results, including an appropriate disclosure of uncertainty for any predictions they make. Level 5 provides a prescription on how to use these predictions as part of a structured management decision process. This requires more communication with management and affected parties to explain the methods, biological rationale and uncertainty, as well as the strengths and limitations of the science and analytical techniques. Our group has had success in integrating scientific advice on environmental impacts into fisheries management decisions at this level through additional model performance evaluations and continual communication and engagement with managers and other interested parties (see ‘*Communication*’ section below). To get to this point, scientists and managers worked together to create a management prescription to outline how scientific advice on the impacts of water temperature and discharge on sockeye salmon mortality (in the form of model predictions) can be used in harvest planning. For Fraser River sockeye salmon, this involved adjusting harvest plans pre-season, using long-range forecasts of summer water temperatures and discharges in in-river loss models (Patterson and Hague, 2007) and, in-season, using forecasts of water temperature and discharge in similar in-river loss models (Macdonald *et al*., 2010). The biological rationale required to convince managers and educate user groups to support the use of these models, the harvest outcomes of which can have major financial and social consequences, is based in large part on physiological research. The totality of research used to support the numerical models includes >30 technical reports and >60 primary publications, the majority of which include physiology to seek mechanistic understandings of temperature- and discharge-related mortality (reviewed by Hinch *et al*., 2012; Johnson *et al*., 2012). Researchers were encouraged by managers to share physiological research and numerical modelling results with representatives of recreational, commercial, First Nations and conservation groups to facilitate acceptance of science-based prescription. All groups interested in the process were made aware of the research, had an opportunity to comment on preliminary results, and provided constructive feedback on new research ideas. When final decisions regarding harvest were being made, the research behind those decisions was not a surprise to those people who would be impacted by the harvest changes. Science is but one source of advice that managers will use in making choices (Rice, 2011); arguably, it should be the most transparent and repeatable.

## Part II: successes and challenges of advancing physiology-based advice

There is a long history of researchers providing descriptive physiological results to management to help explain the impact of factors other than water temperature and discharge on the in-river mortality for Fraser River sockeye salmon. Early work that pre-dates our group includes field studies and laboratory experiments that examined physiological aspects of migratory difficulty and energy allocation (Gilhousen, 1980), cumulative stress (Fagerlund *et al*., 1995), disease progression (Colgrove and Wood, 1966) and suspended sediments (Servizi and Martens, 1987). In this section, we provide examples of our research group using physiological research on pathogens, sex and capture stress to gain a better understanding of in-river mortality in order to elucidate the challenges and successes in converting this type of work into advice for management.

### Pathogens

The role of pathogens has long been associated with in-river mortality of sockeye salmon (Williams, 1973; St Hilaire *et al*., 2002; Jones *et al*., 2003; Miller *et al*., 2014). Early histological examinations of sockeye salmon that died prematurely in the river found a suite of different pathogens that vary annually (Wood, 1965; Williams, 1973), making it difficult to link a specific pathogen to the cause of death. Our more recent work on matching histopathology with host physiological response has provided a better understanding of the mortality associated with some pathogens (Wagner *et al*., 2005; Crossin *et al*., 2008; Bradford *et al*., 2010). In addition, we have started to examine transcriptional responses of sockeye salmon to different types of potential pathogens. The results from these studies have shown that genomic signatures associated with an immune response have the potential to predict migratory failure (Miller *et al*., 2009, 2011). The overall scientific advice from this work has confirmed the potentially important role that pathogens and associated diseases can play in accounting for in-river mortality, but the real utility for management has stalled at this descriptive stage. We are not yet in a position to recommend using pathogen loads, blood chemistry or gene expression patterns to predict fate at the population level for returning wild sockeye salmon given the high uncertainty (i.e. low overall variance in survival explained) in the results (Cooke *et al*., 2006; Miller *et al*., 2011). For example, in the study by Hammill *et al*. (2012), individuals from one of the three populations examined had a different suite of gene expression profiles that could predict fate. In order to potentially progress beyond Level 3 scientific advice, more research is being conducted using novel genomic approaches (e.g. Evans *et al*., 2011; Miller *et al*., 2014) to elucidate stock-specific differences in disease susceptibility that might arise from genetic and environmental influences, distance to the spawning grounds and the probability of pathogen exposure in order to reduce the high uncertainty associated with episodic disease events. More thought is being given by managers with respect to how these results could be used in management, presuming the uncertainty can be reduced. At this time, management acknowledges that there is a physiological explanation for disease-related mortality, but the utility of this scientific advice for predictive purposes will be contingent on the success of planned future work.

### Sex differences

We are closer to making useful predictions for management in our next example, sex-specific mortality patterns. A common observation in our years of research examining factors related to in-river mortality is that female sockeye salmon suffer higher rates of mortality than males in response to stress (e.g. Patterson *et al*., 2004; Gale *et al*., 2014). The evidence for a sex bias is based on both laboratory holding studies and field telemetry studies for which we were able to document sex. In many of the holding studies, the mortality of females was twice that of males (Crossin *et al*., 2008; Gale *et al*., 2011; Robinson *et al*., 2013). The differences detected in the field tagging studies were not as large, but the mortality spread did become magnified with elevated water temperatures (Martins *et al*., 2012). Many of these studies also had supporting physiological measures that provided some potential mechanistic link to the observed differences in survival. Based on a combination of empirical data on sex-specific differences in mortality rates and physiological support in describing this mortality, we were confident in informing management that adult female sockeye salmon die at a higher rate than males in stressful conditions. As such, we unilaterally moved (i.e. without management support) from Level 3 scientific advice to work on population-level predictions consistent with our individual descriptive research.

Unfortunately, the development of any predictive models using sex-specific mortality has stalled at Level 4 because of problems with both the science and management. From the scientific perspective, the patterns of differential mortality reported in our telemetric and holding studies were not reflected in the standard Fisheries and Oceans Canada (DFO; department responsible for managing Pacific salmon in Canada) annual stock assessment spawning ground enumeration studies, assuming a 50:50 ratio for salmon starting the spawning migration; however, annual information on variation in sex ratios of adults returning to the lower river are limited (Foerster, 1968). The ratios of male to female spawners do not appear to vary as a function of high migration temperatures as predicted by our research; the ratios only seemed to vary as a function of extreme high discharge years (Macdonald, 2000; D. Patterson, DFO, unpublished data). Hence, scaling up from research using individuals to the population-level responses did not occur. Moreover, for management, there is no clear way to use the sex-based differences without changing the management objectives. The present goals for achieving spawner escapements for Fraser River sockeye salmon are neutral to sex (i.e. no mention of sex-specific goals in the Pacific Salmon Treaty). We are now left with simply providing suggestions to both our peers and managers on how to proceed further with developing predictive models based on sex-based differences in survival. Researchers need to look more closely at the sex-ratio information collected on the spawning grounds and in downstream fisheries to determine the statistical power to detect varying levels of differential survival (i.e. effect size) given the current fisheries assessment methods. This is likely to be a common challenge in physiological research, because stock assessment information may not be collected at a sufficient level of precision to match the ability to detect a predicted response. More work is needed on the part of physiologists to determine whether experimenter effects of holding or tagging fish are confounding the survival estimates for females. Managers could also re-examine changes to the spawner goals that include female-specific targets. This example has shown that not all research will be useful to management immediately, and there is a risk in moving from Level 3 to Level 4 scientific advice if it is not done with close collaboration of science and management (Fig. 2).

### Fisheries interactions

Our next example uses capture-and-release mortality research to show a more direct connection between the researchers and managers in moving advice from Level 3 to our current attempts to provide Level 5 scientific advice. During the past 10 years, we have focused a large portion of our efforts on understanding the fate of Pacific salmon released after capture, using physiology to elucidate the mechanisms behind lethal and sub-lethal responses (Raby *et al*., 2012, 2015a, b). Major findings from this work include the following: the functional basis for the differences in mortality associated with different gear types (Donaldson *et al*., 2011, 2012, 2013); the role of injury in causing physiological stress and mortality (Nguyen *et al*., 2014); the mixed benefits of using recovery methods (Donaldson *et al*., 2013; Robinson *et al*., 2013; Raby *et al*., 2015c); the among-population variation in mortality responses (Donaldson *et al*., 2012; Robinson *et al*., 2015); the negative impact of high temperature associated with capture and handling (Robinson *et al*., 2013; Gale *et al*., 2014); and the changes in stress responsiveness with maturation stage (Gale *et al*., 2011; Raby *et al*., 2013). All of this work is directly connected to the management problem of trying to describe mortality for post-season accounting of fishing impacts or to predict mortality for harvest planning purposes. The generic Level 3 scientific advice provided to management is that capture-and-release mortality can be understood better through physiology, but it is highly context dependent (Raby *et al*., 2015a), making it challenging to predict.

Predictions of capture-and-release mortality associated with different fishing gear and at different temperatures have been generated based on field telemetry and holding studies that couple survival and physiology. For example, this work has shown that long-term mortality rates for beach-seined and angled sockeye salmon range from 20 to 30% during average summer water temperatures of 18°C (Donaldson *et al*., 2011, 2013). However, mortality will rise rapidly as capture and handling temperatures increase above 19°C, and if they persist above 21°C, there is almost 100% mortality within 4 days (Gale *et al*., 2011; Robinson *et al*., 2013). This advice has been presented to managers for potential use in harvest planning. As with research on thermal impacts on in-river mortality, there is a clear avenue where scientific advice on post-release mortality can be used by management. Each sockeye salmon fishery on the Fraser River has a post-release mortality rate value based on gear type and location (Fisheries and Oceans Canada, 2013). Therefore, management could update their post-release mortality estimates using scientific advice for the different sockeye salmon fisheries, warranting the transition from Level 4 to Level 5 scientific advice.

The time line for managers and researchers to develop a management prescription and start applying new scientific advice at Level 5 will vary. The new research behind the post-release mortality rates for sockeye salmon (reviewed by Raby *et al*., 2015a) has not yet become a part of the management prescription. In this example, the reasons for delay are related to institutional caution and research uncertainty. Institutional resistance to change is common; managers can be cautious when faced with new information and may invoke processes to obtain feedback beyond science before proceeding (Young *et al*., 2013). Furthermore, there is still large uncertainty in the estimates we have derived, and the new mortality rates are, in some cases, considerably higher than those currently used. The higher rates are due, in large part, to the fact that our values are calculated using a longer period for monitoring mortality. The current rates used by management are typically based on 24 h post-release monitoring periods, compared with our estimates that are based on at least 96 h of post-release monitoring. This longer monitoring duration was part of a recent request by fisheries managers to include delayed mortality associated with fishing interactions. The current plan for incorporating new information as official scientific advice will require further meetings with various stakeholders and First Nations.

As part of this plan for advancing the research on catch-and-release mortality, there is a formal request from the fisheries management sector to the science sector of DFO to develop scientific advice on updating post-release mortality rates using relatively new research. In Canada, we have a formal mechanism under the Canadian Science Advisory Secretariat that allows fisheries management to request formal scientific advice from their own agency. Our research team has been commissioned by the Canadian Science Advisory Secretariat to write the research document that will be the basis for this advice. The document will involve an in-depth look at the mechanistic (i.e. physiological) basis for how different factors impact fishing-related mortality, as well as a review of the mortality rates themselves. In other words, the official scientific advice will be based in large part on the ability of physiologists to explain why certain factors, such as injury, air exposure, handling time, capture time and revival methods, are important in generating estimates of fishing-related mortality (reviewed by Raby *et al*., 2015a). This information will be shared with all groups with a vested interest in the salmon fishery. Changes to mortality rates will not be made until all groups have had an opportunity to comment and process the new information; underscoring the central role that communication plays in any plan that provides scientific advice to management.

## Communication

Scientific advice is not being given in a vacuum; meaning that this information will disseminate beyond the initial management audience, and therefore, it is important to be cognisant of the interpretation and use of this information by different groups. A communication plan is likely to be essential for success at the fifth level, but ideally it should be initiated the first time that managers and researches begin to exchange information and be the responsibility of both managers and researchers. The first priority for communicating research results is to satisfy management requirements, but researchers cannot be naïve to how other groups, such as the media, environmental groups, scientific community and fishing groups, can accidently misinterpret or deliberately spin the results. Misrepresentations can potentially derail the ability of managers to use physiological research, as exemplified earlier with the focus on upper lethal thermal limits (Servizi and Jensen, 1977), because they typically rely on information and feedback from these same outside groups to help make decisions (e.g. Cohen, 2012). It is worth repeating that scientific advice is only one source of information that managers rely on to make decisions (Rice, 2011). It is the responsibility of researchers to communicate clearly both the strengths and the limitations of the research to managers and a broader audience. In practical terms, this means a full disclosure of the uncertainty in the results, as well as clearly stating how the research does and/or does not relate to management or any other issue a stakeholder may decide to link it to (Regan *et al*., 2002; Harwood and Stokes, 2003). Managers, in turn, need to review the work critically, anticipate future criticisms and prepare researchers for stakeholder responses to their work. Unfortunately, the primary media used by researchers for communicating science (peer-reviewed journal articles) are likely to be one of the least effective or desirable methods for communicating this information to either management or other interested groups (Nguyen *et al*., 2012). Increasing the impact and relevance of physiological research will require not only good science but also effective communication with management and other interested parties.

## Synopsis

The reasons for the success of our work include political motivation, funding, accountability, legal clarity, institutional environment, personal relationships and peer acceptance. For example, fisheries management recognized the potential problem of temperature-based in-river loss after a series of high-profile reviews in the early 1990s (Larkin, 1992; Fraser, 1995) and again in the early 2000s (Canadian Standing Committee on Fisheries and Oceans, 2005; Williams, 2005). Each of these government-commissioned reviews led to an increase in funding for sockeye salmon research on problems related to in-river mortality. Our research group benefited from the direct connection between a management problem and funding of physiologically based solutions. This funding included opportunities for traditional national science funds (e.g. Natural Sciences and Engineering Research Council of Canada; NSERC), strategic national science funds (e.g. NSERC-Strategic and NSERC-Network), internal government science agency funds, and competitive applied research funds from the Canada–US Pacific Salmon Treaty. Researchers were then held accountable to report both to their academic peers, through publications in scientific journals, and to the funding agencies via management. This dual accountability produced quality science that was also applied in nature. The legal framework of the Pacific Salmon Treaty also provided clarity of objectives and clear mechanisms to incorporate mitigation measures based on environmental impacts. Likewise, the post-release mortality rates in the official fishery planning documents of DFO (Fisheries and Oceans Canada, 2013) provide a clear outlet for using research results from the fisheries interaction work. The institutional environments of both groups also helped. The Canadian Science Advisory Secretariat process provides a direct link between scientific research, scientific advice and fisheries management. Management agencies also invite researchers to speak at annual meetings and organize strategic workshops on critical management issues. Our research group is also proactive and continues to hold annual workshops with managers, fishing sector representatives, First Nations and conservation groups (e.g. Hinch and Gardner, 2009) to encourage feedback. The benefits of these interactions are twofold. First, the people most affected by decisions that use our scientific advice were familiar with the research prior to it being used by management. Second, it fostered improved personal relationships among all groups, which improved overall trust, a key element in knowledge transfer (Young *et al*., 2013). The role that individual relationships can have to ensure success cannot be underestimated; this includes access to experts with the diverse skills required to tackle complex science questions. We were fortunate to know and be able to work with managers, ecologists, numeric modellers and physiologists. This collaboration has produced physiology publications that have been recognized by researchers internationally as major scientific contributions in the field of applied genomics (e.g. Miller *et al*., 2011) and oxygen limitation theory (Eliason *et al*., 2011). The scientific peer acceptance along with the institutional and situational conditions has been helpful in gaining support from management.

The major challenges to providing scientific advice beyond Level 3, the simple descriptions of nature, are rooted in the different demands of science and management. Scientists are rewarded based in large part on developing novel scientific explanations for how the world works (Knorr Cetina, 1999; Provencal, 2011). Therefore, the interest of the researcher peaks at Level 3, where they can maximize the number of novel publications of different factors that influence survival in fish. Many researchers feel that simply by providing a scientific explanation for a specific management problem, they have completed their obligation to show the applied nature of their work (Blackman and Benson, 2012). They are often unaware of the extra work required to make confident predictions regarding future outcomes tailored to objectives of a specific fishery; this work requires more research and collaboration to reduce and quantify uncertainty to a level that managers or other knowledge practitioners want (Mergel *et al*., 2008). There is rarely any career advantage or reward for taking this path (Young *et al*., 2013). Conversely, managers must make decisions in a timely manner, and they are not specifically rewarded for knowing why a particular outcome has occurred, only that it is working. Therefore, the interest of managers peaks at Level 5, when there is a clear plan to use the information to aid in management. There is often no obligation, no accountability or no reward for managers that actively seek scientific advice at Level 3 and help to develop this research into Level 5 scientific advice. For the process to be effective, the researcher needs to put the management problem in the matrix of what is already known and unknown before consuming more time and money. For example, a synthesis of the existing knowledge base can be made to determine the level of uncertainty in management outcomes that will be reduced under different research projects. It is important to recognize that all of the advice provided, even at Level 1, is building a connection and trust among managers and researchers. Therefore, if a researcher is asked by management to provide scientific advice, it is important to do so expeditiously, along with a healthy dose of uncertainty (see Regan *et al*., 2002), before asking for funds and deferring an answer until they have the perfect descriptive or predictive model. Managers often need to act before scientific consensus is achieved (Ludwig *et al*., 1993). Likewise, researchers walking away from the problem after they have published a few descriptive papers on a subject will not go far in getting their science to be effective in management, given the large gap between Level 3 and Level 5. Managers and researchers alike could also benefit from seeing examples of where the long-term benefit of scientific advice at different levels has led to better management. There is lack of retrospective evaluations of the efficacy of scientific advice to improve management performance in fisheries.

The temperature- and discharge-based mortality models are an exception in that we have performed retrospective evaluations of model performance at the behest of management (Cummings *et al*., 2011). We have also learnt from the development of the harvest adjustment models that most of these efforts will cost hundreds of thousands of dollars, take years of sustained effort to complete, use a team of researchers willing to meet with managers and stakeholders, require continuous model development and refinement and require extensive communications in the form of primary publications, technical reports, briefing notes, management meetings and presentations for public engagement, before management will have an updated prescription process to use new information supported by the physiological research. This is not meant as criticism of either side, but simply the reality of getting your specific prescription to market. Science will always be only a part of the decision-making process, but how big a part—the level of advice it can achieve—will depend on people, managers and researchers alike, who can help to promote the use of conservation physiology.
